# Cardiovascular Disease Awareness and Health Among Healthcare Workers and the General Population in Bangalore, India: A Comparative Multivariable Analysis

**DOI:** 10.7759/cureus.95180

**Published:** 2025-10-22

**Authors:** Rashmi Venkatesh, Siya Patel, Stan Grogg

**Affiliations:** 1 Public Health, William Carey University College of Osteopathic Medicine, Hattiesburg, USA; 2 Public Health, Oklahoma State University Center for Health Sciences, Tulsa, USA

**Keywords:** awareness, cardiovascular disease, global health, physical health, public health

## Abstract

Introduction: Cardiovascular disease (CVD) has become a critical public health challenge in India, with a steadily rising burden observed over the past few decades. Notably, the Indian population tends to develop cardiovascular conditions at a younger age compared to other regions. In Bangalore, preliminary studies suggest that baseline knowledge among healthcare workers may still be lacking, and younger populations, including college students and nursing trainees, often demonstrate inconsistent understanding of cardiac risk factors, particularly around nutrition and disease mechanisms. Methods: This cross-sectional study assessed knowledge of cardiac disease and self-reported physical health among the general population and healthcare workers in Bangalore, Karnataka. A survey of 11 questions was adapted from previously validated questionnaires on CVD awareness and health behaviors. Participants were recruited through convenience sampling for healthcare workers and snowball sampling for the general population. For the general population, the survey was disseminated through an online form in Qualtrics, with initial respondents encouraged to share the link to reach a broader audience. Healthcare workers, students, and adjacent staff were recruited from the Sir C.V. Raman Hospital and Rehoboth Nursing School in Bangalore, where paper versions of the survey were distributed. The survey responses were analyzed, and data preprocessing included cleaning the dataset, excluding individuals under the age of 18 or over 65, and recoding categorical variables into binary formats. Chi-square tests were conducted to assess significant differences between healthcare workers and the general population in their survey responses. A p-value of less than 0.05 was considered indicative of statistical significance.

Results: From the 152 total responses, participant demographics consisted of healthcare professionals who were predominantly female students aged 18-24, in comparison to the general population, which was mainly individuals aged 45-54. Concerning CVD knowledge and awareness, a significant difference was observed, in which 46 (88.5%) respondents of the general population correctly identified that cholesterol should be checked every one to three years, which differed from the 69 (70.1%) respondents from the healthcare professionals' group who correctly identified the answer. In the assessment of one's own health, 31 (47.7%) participants of healthcare professionals reported falling ill more than the average person, in contrast to the 10 (25%) participants of the general population. Physical activity levels were also higher in the general population, with 24 (50%) of the group exercising more than 120 minutes per week, compared to just 10 (10.4%) participants from the healthcare professionals' group.

Conclusion: The disparity in the results regarding cardiovascular health knowledge between healthcare workers and the general population raises concerns about a possible gap in current healthcare education. Our study was able to add to the current literature surrounding both the current knowledge base regarding cardiovascular health in Bangalore and how medical and health knowledge does not equate to healthier lifestyle practices among healthcare workers.

## Introduction

Cardiovascular disease (CVD) has become a critical public health challenge in India, with a steadily rising burden observed over the past few decades. According to data from the Global Burden of Disease Study, India has experienced a significant epidemiological shift, with non-communicable diseases, such as CVD, now accounting for the majority of morbidity and mortality [1-3]. Notably, the Indian population tends to develop cardiovascular conditions at a younger age compared to other regions, and substantial knowledge gaps persist regarding disease risk factors and preventive strategies, especially in rural and underserved areas [1,4-5]. 

In 2015, a landmark study was conducted, where a comprehensive systemic review spanning across 40 years’ worth of coronary artery disease research seeking to explore the risk factors and treatment of coronary disease in Gujarat, India, conveying that, despite ongoing research, the Indian population continues to face widespread exposure to established CVD risk factors such as hypertension, hypercholesterolemia, diabetes, and smoking [6-8]. These risk factors are well-documented, yet awareness of their link to heart disease remains suboptimal in many communities [9]. A recent 2023 review reaffirmed this trend, highlighting the role of biological predispositions, including lipid and glucose metabolism and lifestyle patterns-particularly sedentary behavior and poor nutrition, in accelerating disease onset [10-12]. The challenge, therefore, lies not only in understanding CVD prevalence but also in identifying the extent of awareness about modifiable risk factors among different segments of the population. 

While studies have explored cardiovascular risk awareness in select Indian regions, there remains a relative paucity of comparative data examining knowledge disparities between healthcare professionals and the general public, especially within individual urban settings. In Bangalore, one of India’s fastest-growing metropolitan cities, preliminary studies suggest that while educational interventions via lectures with PowerPoint aids and providing information and awareness concerning lifestyle factors influencing coronary artery disease, with topics including tobacco use, dietary modifications, and methods to attenuate stress can improve knowledge and behavioral practices among high-risk individuals [13], baseline knowledge, even among healthcare workers, may still be lacking [14]. Moreover, younger populations, including college students and nursing trainees, often demonstrate inconsistent understanding of cardiac risk factors, particularly around nutrition and disease mechanisms [14-15]. 

This study seeks to build upon existing literature by investigating cardiovascular health awareness in Bangalore, Karnataka, among both healthcare professionals and the general population. The dual focus allows for a comparative analysis that may reveal critical gaps in public and professional knowledge alike. Previous region-specific studies have emphasized the high burden of cardiovascular risk in Bangalore [16]. A 2022 cross sectional study assessed CVD risk among patients at a local hospital in Bangalore, Karnataka, and it was subsequently concluded that 14% of the study population had high risk versus and an addition of 20% having moderate-high CVD risk [16], yet few have addressed how informed its diverse residents are about the disease’s causes, symptoms, and preventive measures. By leveraging validated survey tools and region-specific data, this study aims to identify knowledge disparities that could inform future educational interventions and public health strategies across similar urban populations in India.

## Materials and methods

This cross-sectional study assessed knowledge of cardiac disease and self-reported physical health among the general population and healthcare workers in Bangalore, Karnataka. A survey was constructed with 11 questions, all of which were adapted from previously validated questionnaires on CVD awareness and health behaviors (see Appendix) [17-19]. All validated questionnaires were of open access and did not require permission for use. Participants were recruited through convenience sampling for 99 healthcare workers and snowball sampling for the 53 general population participants. For the general population, the survey was disseminated through an online form in Qualtrics, with initial respondents encouraged to share the link to reach a broader audience. Healthcare workers, students, and adjacent staff were recruited from the Sir C.V. Raman Hospital and Rehoboth Nursing School in Bangalore, where paper versions of the survey were distributed with the help of Power of a Nickel (PON), a non-profit, medical outreach program, which provides care for patients across many countries. Through collaboration with local organizations, PON works towards building relationships within communities, supplying medical care to the underserved, and promoting health education. With their assistance, the dissemination of the survey was conducted.

The survey responses were entered into IBM SPSS Statistics for Windows, Version 29.0 (released 2022, IBM Corp., Armonk, NY) for analysis. Data preprocessing included cleaning the dataset, excluding individuals under the age of 18 or over 65, and recoding categorical variables into binary formats. Chi-square tests were conducted to assess significant differences between healthcare workers and the general population in their survey responses. A p-value of less than 0.05 was considered indicative of statistical significance. This study was approved by the Institutional Review Board (IRB) at William Carey University in November 2024 (ID #2024-065). Participants provided informed consent before completing the survey, and all responses were collected without collecting personal identifiers to ensure confidentiality.

## Results

Demographic characteristics of the two groups, healthcare professionals and the general population, surveyed to determine their cardiovascular awareness, can be found in Table 1. There was a higher proportion of female participants among healthcare professionals compared to males. The majority of healthcare professionals were students aged 18-24 years, whereas most participants from the general population were aged 45-54 years and employed. In addition, 48 (50.0%) respondents from the healthcare professionals' group were enrolled in college but had not yet graduated, while 38 (71.7%) individuals of the general population had completed their education, including some form of professional schooling. 

**Table 1:** Demographic characteristics of the survey participants: healthcare professionals (n = 99) versus the general population (n = 53)

Variable	Response	Healthcare professionals	General population
		99 (65.1)	53 (34.9)
Gender	Male	19 (19.4)	29 (55.8)
	Female	79 (80.6)	23 (44.2)
Age (years)	18-24	72 (72.7)	11 (20.8)
	25-34	11 (11.1)	2 (3.8)
	35-44	5 (5.1)	3 (5.7)
	45-54	4 (4.0)	23 (43.4)
	55-64	7 (7.1)	14 (26.4)
Level of Education	SSC (Secondary School Certificate) and HSC Higher Secondary Certificate	16 (16.7)	0 (0.0)
	Some college but not a graduate	48 (50.0)	2 (3.8)
	Graduate/postgraduate (general)	12 (12.5)	13 (24.5)
	Graduate/postgraduate (professional)	20 (20.8)	38 (71.7)
Employment status	Employed	28 (28.30)	38 (71.7)
	Unemployed	2 (2.0)	1 (1.9)
	Student	69 (69.7)	3 (5.7)
	Homemaker	0 (0.0)	7 (13.2)
	Retired	0 (0.0)	4 (7.5)

Table 2 demonstrates the responses to how often one’s cholesterol should be checked between healthcare workers and the general population. A significant difference was observed between the two groups, with 68 (70.1%) healthcare workers correctly answering that cholesterol should be checked every one to three years in comparison to the general population, where 46 (88.5%) selected the correct answer choice. 

**Table 2:** Association of cholesterol surveillance knowledge and participant group: healthcare professionals versus general population (n = 149)

Question and response		Healthcare workers (n = 97)	General population (n = 52)	Chi-square	P-value
How often should cholesterol be checked?	Incorrectly answered	29 (29.9)	6 (11.5)	6.348	0.012
	Correctly answered that cholesterol should be checked every one to three years	68 (70.1)	46 (88.5)		

In a question that aimed to evaluate participants’ assessment of their overall health, notable differences emerged between healthcare workers and members of the general population. Nearly half of the healthcare workers surveyed at 31 (47.7%) participants agreed with the statement that they tend to fall ill more frequently than the average person. By contrast, only 10 (25%) respondents from the general population endorsed this statement, with a substantial majority being 30 (75%) respondents disagreeing. 

**Table 3:** Association of personal health perception and participant group: healthcare professionals versus the general population (n = 105)

Question and response		Healthcare workers (n = 65)	General population (n = 40)	Chi-square	P-value
How TRUE or FALSE is each of the following statements for you? I seem to get sick a little easier than other people.	False	34 (52.3)	30 (75.0)	5.357	0.021
	True	31 (47.7)	10 (25.0)		

Table 4 presents the reported levels of physical activity, measured by the number of minutes of exercise completed per week, comparing healthcare professionals and the general population. While only 10 (10.4%) individuals from the healthcare professionals group reported engaging in more than 120 minutes of exercise per week, 24 (50%) individuals in the general population met or exceeded this level of activity.

**Table 4:** Association of activity levels and participant group: healthcare professionals versus the general population (n = 144)

Question and response		Healthcare workers (n = 96)	General population (n = 48)	Chi-square	P-value
During the past 4 weeks, on average, how many minutes of exercise per week have you completed?	Less than or upto 120 minutes	86 (89.6)	24 (50.0)	27.799	< .001>
	>120 minutes	10 (10.4)	24 (50.0)		

## Discussion

Of the questions that pertained to assessing knowledge regarding cardiac disease and prevention, only one question was identified in which there were significant differences between the healthcare workers and the general population. Surprisingly, a greater percentage of the healthcare workers incorrectly answered the question, “how often should cholesterol be checked,” at 20 (29.9%) participants in comparison to the general population, with six (11.5%) participants. The disparity in the results could be attributed to several factors. First, being that over half of the healthcare workers are nursing students who have not received their full medical education yet. This raises the concern for a possible gap in current healthcare education regarding preventive medicine. If students carry these misunderstandings into practice, this could potentially increase the risk for not only inadequate levels of screening for cardiovascular disease but also lead to missed opportunities in implementing preventative measures as well. However, it must also be noted that the question itself could have been written better to ensure complete clarity and eliminate any potential confusion among participants. While all questions were selected from previous cardiovascular surveys, modification of the answer choices could have been made prior to survey dissemination. As it currently stands, the US Preventive Services Task Force (USPSTF) delineates the need for lipid screening on the basis of cardiovascular risk scores. As a result, the frequency for cholesterol screening increases when risk is high (one to three years), and individuals with less risk do not need to be evaluated as often (three to five years). A question that takes into consideration cardiovascular risk with regard to lipid screening could have potentially ameliorated confusion among students. 

When examining the questions that assessed participants' views on their own current health, there were significant differences between the healthcare workers and the general population. Specifically, healthcare workers reported having a worse perception of their own health, with 34 (52.3%) respondents agreeing to the statement that they get sick more often than other people, as well as almost 90% of respondents reporting less than 120 minutes of exercise each week. Healthcare professionals' poor perception of their own health is consistent with a 2024 study conducted in Bangalore, which illustrated high rates of burnout among nursing students [20]. These findings parallel what is seen among healthcare professionals in the United States, in which current literature reports healthcare workers being at higher risk for a variety of different ailments, ranging from sleep and musculoskeletal disorders to depression, anxiety, and burnout [21]. In fact, studies have elucidated poor compliance with healthy lifestyle recommendations among nurses and physicians [22]. While doctors are in agreement that diet and exercise are imperative for good health, only 25% were found to adhere to these guidelines themselves, according to research [22]. Nurses also report poor health habits, including smoking, alcohol use, and sedentary lifestyles [22-23]. 

There are a few limitations to this study that must be accounted for as well. Within the healthcare workers' data, 79 (80.6%) participants were female. In addition, as discussed above, 59 of the 99 (59.6%) respondents were nursing school students. The overall composite of healthcare worker participants was female nursing students. The study’s sample size also presents as a limitation, with 152 participants. The smaller sample size restricts the overall generalizability of the study results to the Bangalore population. For future research, a larger cohort is warranted to improve upon the external validity. 

## Conclusions

These results offer several different avenues of research worth further pursuing with larger and more diverse sample sizes. Our study was able to add to current literature surrounding the health of physicians and healthcare workers, in which medical and health knowledge does not equate to healthier lifestyle practices. Further exploration of this dichotomy is warranted in efforts to not only be able to draw similarities among healthcare workers on a global scale but also to reform the current medical curriculum and create interventions that would aim to incorporate self-care and healthy lifestyle strategies into medical training. 

## Appendices

**Table 5:** Cardiac Awareness Questionnaire

Section	Question/statement	Response options
Statements about heart diseases	Smoking is a risk factor.	True ☐ False ☐
	High blood pressure is a risk factor.	True ☐ False ☐
	High cholesterol is a risk factor.	True ☐ False ☐
	Being overweight increases the risk of heart disease.	True ☐ False ☐
	Family history increases the risk of heart disease.	True ☐ False ☐
	How often should blood pressure be checked?	☐ Every year ☐ Every 3 years ☐ Every 5 years ☐ I don’t know
	How often should cholesterol be checked?	☐ Every 1–3 years ☐ Every 3–5 years ☐ Every 6–10 years ☐ I don’t know
Preventative health	The doctor has discussed smoking, diet, and exercise with me.	☐ Definitely False ☐ Mostly False ☐ Not Sure ☐ Mostly True ☐ Definitely True
	I have received cardiac information from my doctor.	☐ Definitely False ☐ Mostly False ☐ Not Sure ☐ Mostly True ☐ Definitely True
	I know where to go to get preventative tests for cardiac disease.	☐ Definitely False ☐ Mostly False ☐ Not Sure ☐ Mostly True ☐ Definitely True
	How would you rate your knowledge about preventative health measures?	☐ Excellent ☐ Very Good ☐ Good ☐ Fair ☐ Poor
	How would you rate your general health?	☐ Excellent ☐ Very Good ☐ Good ☐ Fair ☐ Poor
	During the past four weeks, how much did physical health limit your activities?	☐ Not at all ☐ Very Little ☐ Somewhat ☐ Quite a Lot
	How much bodily pain have you experienced?	☐ None ☐ Very Mild ☐ Mild ☐ Moderate ☐ Severe ☐ Very Severe
	Did pain interfere with your normal work?	☐ Not at all ☐ A little bit ☐ Moderately ☐ Quite a bit ☐ Extremely
Statements about own health	I get sick more easily than others.	☐ Definitely False ☐ Mostly False ☐ Not Sure ☐ Mostly True ☐ Definitely True
	I am as healthy as anybody.	☐ Definitely False ☐ Mostly False ☐ Not Sure ☐ Mostly True ☐ Definitely True
	I expect my health to get worse.	☐ Definitely False ☐ Mostly False ☐ Not Sure ☐ Mostly True ☐ Definitely True
	Minutes of exercise per week (past four weeks).	☐ 0 minutes ☐ 5–30 minutes ☐ 30–60 minutes ☐ 60–90 minutes ☐ 90–120 minutes ☐ >120 minutes
